# The Sustained Delivery of Resveratrol or a Defined Grape Powder Inhibits New Blood Vessel Formation in a Mouse Model of Choroidal Neovascularization

**DOI:** 10.3390/molecules191117578

**Published:** 2014-10-30

**Authors:** Mozhgan Rezaie Kanavi, Soesiawati Darjatmoko, Shoujian Wang, Amir A. Azari, Mitra Farnoodian, Jason D. Kenealey, Paul R. van Ginkel, Daniel M. Albert, Nader Sheibani, Arthur S. Polans

**Affiliations:** 1 Ophthalmic Research Center, Shahid Beheshti University of Medical Sciences, Tehran 1666663111, Iran; E-Mail: rezaeikanavi@gmail.com; 2 Department of Ophthalmology and Visual Sciences, University of Wisconsin, Madison, WI 53792 USA; E-Mails: srdarjat@wisc.edu (S.D.); shoujianwang@wisc.edu (S.W.); farnoodian@wisc.edu (M.F.); prvangin@wisc.edu (P.R.G.); dalbert@wisc.edu (D.M.A.); nsheibanikar@wisc.edu (N.S.); 3 Wills Eye Hospital, Philadelphia, PA 19107, USA; E-Mail: amirazarimd@gmail.com; 4 Department of Nutrition, Dietetics and Food Science, Brigham Young University, Provo, UT 84602, USA; E-Mail: jason_kenealey@byu.edu

**Keywords:** age-related macular degeneration, choroidal neovascularization, angiogenesis, natural products, resveratrol, grape constituents

## Abstract

The objective of this study was to determine whether resveratrol or a defined, reconstituted grape powder can attenuate the formation of new blood vessels in a mouse model of choroidal neovascularization (CNV). To accomplish this objective, C57BL/6J mice were randomized into control or treatment groups which received either resveratrol or grape powder by daily oral gavage, resveratrol or grape powder delivered ad libitum through the drinking water, or resveratrol by slow release via implanted osmotic pumps. A laser was used to rupture Bruch’s membrane to induce CNV which was then detected in sclerochoroidal eyecups stained with antibodies against intercellular adhesion molecule-2. CNV area was measured using fluorescence microscopy and Image J software. *Ad libitum* delivery of both resveratrol and grape powder was shown to significantly reduce the extent of CNV by 68% and 57%, respectively. Parallel experiments conducted *in vitro* demonstrated that resveratrol activates p53 and inactivates Akt/protein kinase B in choroidal endothelial cells, contributing to its anti-proliferative and anti-migratory properties. In addition resveratrol was shown to inhibit the formation of endothelial cell networks, augmenting its overall anti-angiogenic effects. The non-toxic nature of resveratrol makes it an especially attractive candidate for the prevention and/or treatment of CNV.

## 1. Introduction

Age-related macular degeneration (AMD) is a leading cause of blindness in the elderly, affecting millions of individuals worldwide. Approximately 90% of the vision loss associated with AMD occurs as the consequence of choroidal neovascularization (CNV) [1,2,3,4]. Current treatment strategies for neovascular AMD include the targeting of the pro-angiogenic factor, vascular endothelial growth factor A (VEGF-A) [5,6]. Multiple intravitreal injections of humanized monoclonal anti-VEGF antibodies have allowed greater control over the deregulated angiogenesis associated with CNV [7,8,9]. However, in addition to high treatment cost, repeated intravitreal injections are reportedly associated with risk of endophthalmitis and other complications [9,10,11]. Many patients also are averse to having their eyes perforated with a needle.

Resveratrol (3,5,4'-trihydroxystilbene) is a polyphenol found in many plants consumed in the normal diet such as grapes, berries and peanuts, as well as in other plant sources used as herbal medicines. Resveratrol has attracted a great deal of attention because of its effects on cell signaling pathways relevant to disparate diseases [12,13]. In addition to cardioprotective and neuroprotective properties [14,15,16,17,18,19,20,21], resveratrol has been reported to provide protection against various cancers [22,23,24,25], diabetes [26,27,28], inflammatory diseases [29,30,31,32] and senescence [33,34]. Effects of resveratrol on angiogenesis also have been reported, but its responses are sometimes conflicting [35,36,37]. Resveratrol has been shown to be pro-angiogenic in models of myocardial ischemia [35,38], while it exhibits anti-angiogenic effects in numerous malignancies [39,40,41,42]. Therefore, it appears that resveratrol elicits different effects in diverse angiogenic beds that may reflect the activation of different molecular mechanisms.

Extracts derived from grapes, containing resveratrol, related polyphenols and other bioactive plant products, also have been reported to possess clinically relevant anti-oxidant activities as well as anti-inflammatory, anti-proliferating and gene expression altering capacities *in vitro* [43,44]. Experimental studies further support a protective role of grape seed extracts against reperfusion injury after ischemia in the myocardium, brain and liver [45,46,47,48,49,50]. Like resveratrol, grape extracts can induce or inhibit angiogenesis. They were shown in some reports to support dermal wound healing through up-regulation of VEGF expression, while in other studies they instigated anti-angiogenic actions through down-regulation of VEGF and angiopoietin signaling [51,52,53].

Resveratrol as well as other bioactive components of grapes have the potential of safely modulating pathological angiogenesis associated with different ocular diseases with harmful retinal or choroidal neovascularization [36,54,55,56]. While assessing their angiogenic properties in some models of disease such as cancer may be confounded by the intertwining of multiple cell types and tissues, the eye is an especially useful model system because it contains several vascular beds sandwiched between avascular tissues. Laser-induced choroidal neovascularization for example, can be measured without complications from the sclera and avascular retinal pigment epithelium, and a number of potential anti-angiogenic drugs have been validated using this model. For these reasons, the experimental study presented here was designed to verify whether administration of resveratrol or a defined, reconstituted grape powder has the ability to attenuate neovascularization in a laser-induced mouse model of CNV. Both resveratrol and the reconstituted grape powder are shown in this study to inhibit neovascularization in the laser-induced model of CNV, dependent on their dose and rate of delivery.

The formation of new blood vessels from existing ones, as occurs in CNV, involves the activation of endothelial cells that can degrade the basement membrane to escape the pre-existing vessel, the proliferation of these endothelial cells, their migration to the site of the angiogenic stimulus, and finally the formation of new, immature vessels. *In vitro* studies presented here demonstrate that resveratrol can intervene in several of these steps by inhibiting the proliferation, migration and network formation of activated endothelial cells. Cellular pathways involving p53 and Akt/protein kinase B are shown to be modulated by resveratrol, potentially explaining its anti-proliferative and anti-migratory effects and its overall anti-angiogenic properties.

## 2. Results and Discussion

Following laser injury to Bruch's membrane, an extension of subretinal blood vessels from the choroid, reminiscent of neovascular events associated with the exudative form of AMD, was observed in each of the control eyes in this study. These controls then served as comparisons for treatment groups to assess the efficacy of different drugs as well as different modes of drug delivery.

### 2.1. Adlibitum Intake of Water Containing either Resveratrol or Reconstituted Grape Powder

Resveratrol has limited solubility in aqueous solutions, approximately 100 µg/mL. Mice utilized in this study, consuming about 5 mL of fluid/day, thereby attained a maximum daily intake of approximately 500 µg of resveratrol or a dose equivalent to 25 mg/kg/day. As noted below, this corresponds to only 50% of the saturating concentration of resveratrol that can be achieved daily in a bolus by oral gavage. In contrast to oral gavage, however, the consumption of water containing resveratrol or reconstituted grape powder occurs over the 24 h timeframe of each day.

The concentration of resveratrol in the reconstituted grape powder, 0.7 mg/kg, resulted in the daily intake of approximately 0.07 µg per mouse or a dose equivalent to 3.5 µg/kg/day. However, in addition to resveratrol the grape powder contains high levels of additional bioactive agents, including flavonols such as quercetin (71.3 mg/kg), anthocyanins (458.9 mg/kg) and catechins (46.9 mg/kg), which also can modulate angiogenic activity.

**Figure 1:**
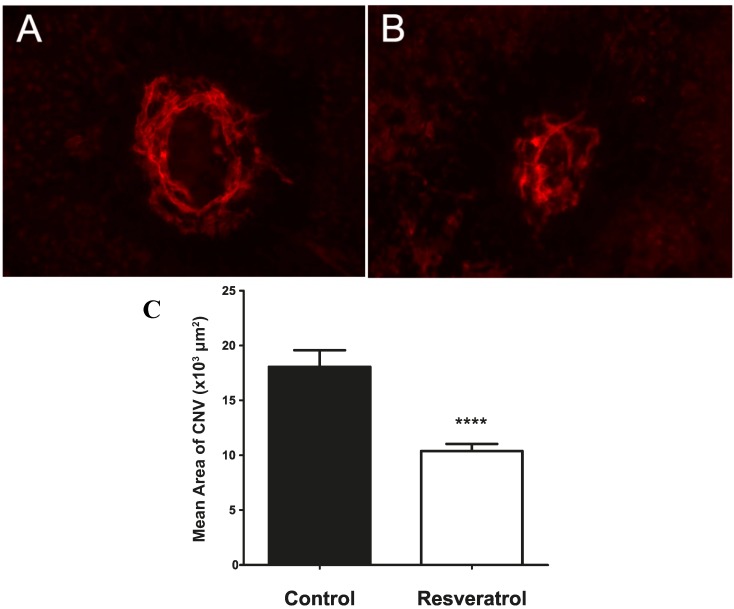
Resveratrol reduces blood vessel formation in a mouse model of CNV. Representative sclerochoroidal flat mounts after staining with intercellular adhesion molecule-2 antibodies; CNV in mice that received vehicle (**A**) or resveratrol (**B**) in their drinking water. Treatment began five days prior to the induction of CNV and continued for a total of 16 days. (**C**) Quantification of the data (**** *p* value resveratrol = 0.0001, n = 5 eyes).

As shown in Figure 1, new blood vessel formation was reduced significantly when animals were treated with resveratrol. Under these conditions of ad libitum water intake, the average CNV area in control animals, 18,052 ± 1516 μm^2^, exceeded the area of blood vessel formation observed in animals treated with resveratrol, 10,378 ± 646 μm^2^. Resveratrol reduced new blood vessel formation by approximately 43%.

**Figure 2:**
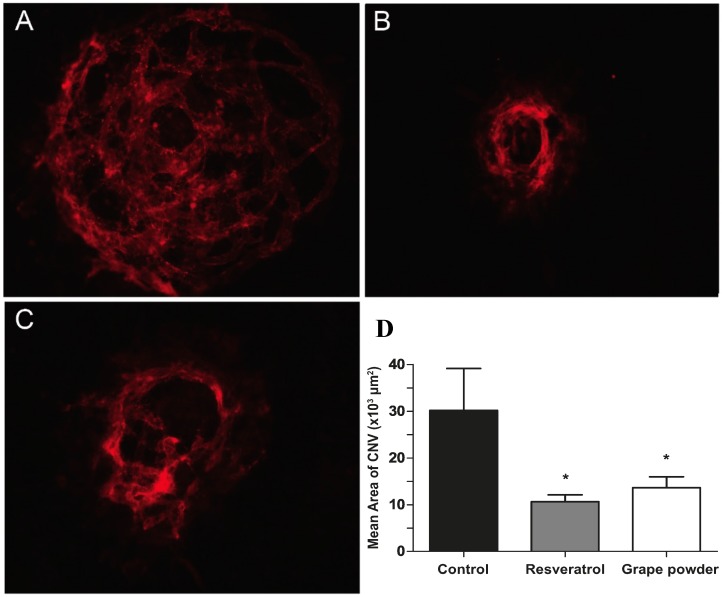
Resveratrol and reconstituted grape powder reduce blood vessel formation in a mouse model of CNV. Representative sclerochoroidal flat mounts after staining with intercellular adhesion molecule-2 antibodies; CNV in mice that received vehicle (**A**), resveratrol (**B**) or reconstituted grape powder (**C**) in their drinking water. Treatment began twelve days prior to the induction of CNV and continued for a total of 24 days. (**D**) Quantification of the data (* *p* value resveratrol = 0.018, n = 5 eyes); * *p* value grape powder = 0.042, n = 5 eyes each).

Extension of the pretreatment period by one week resulted in the further reduction in blood vessel formation. The CNV area in control animals, 30,727 ± 9514 μm^2^ far exceeded the area of new blood vessel formation observed in animals treated either with resveratrol, 9908 ± 1354 μm^2^ or with reconstituted grape powder, 13,380 ± 2442 μm^2^ (Figure 2A–D). Resveratrol and reconstituted grape powder reduced new blood vessel formation by 68% and 57%, respectively.

### 2.2. Bolus Delivery of Resveratrol by Daily Oral Gavage

**Figure 3:**
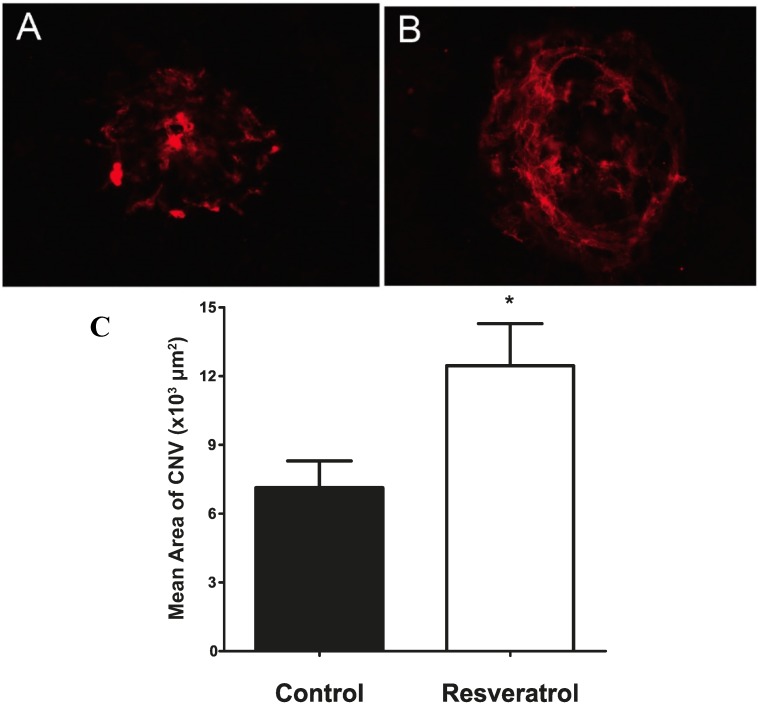
Single daily delivery of resveratrol enhances blood vessel formation in a mouse model of CNV. Representative sclerochoroidal flat mounts from mice receiving vehicle (**A**) or resveratrol (**B**) once daily by oral gavage. Treatment began five days prior to the induction of CNV and continued for a total of 16 days. (**C**) Quantification of the data (* *p* value = 0.023; n = 18 eyes).

The single daily delivery of resveratrol in these experiments was approximately 1 mg/mouse, equating with a dose of 50 mg/kg/day or roughly twice the amount of resveratrol compared to the quantity delivered through the drinking water, as described in the previous experiment. While the delivery of resveratrol under these conditions has been shown to inhibit neovascularization and tumor growth in different mouse models of cancer, it proved insufficient to curtail the growth of new blood vessels in the mouse model of CNV (Figure 3A–C). As illustrated in Figure 3, mice treated by daily oral gavage with resveratrol displayed larger neovascular outgrowth compared to the control animals. An approximately 1.7-fold increase in the average CNV area was observed in the resveratrol-treated group (12,454 ± 1831 µm^2^; n = 18 eyes) compared to the control group (7134 ± 1162 µm^2^; n = 14 eyes). The difference was marginally significant (*p* = 0.023).

### 2.3. Bolus Delivery of Reconstituted Grape Powder by Daily Oral Gavage

**Figure 4:**
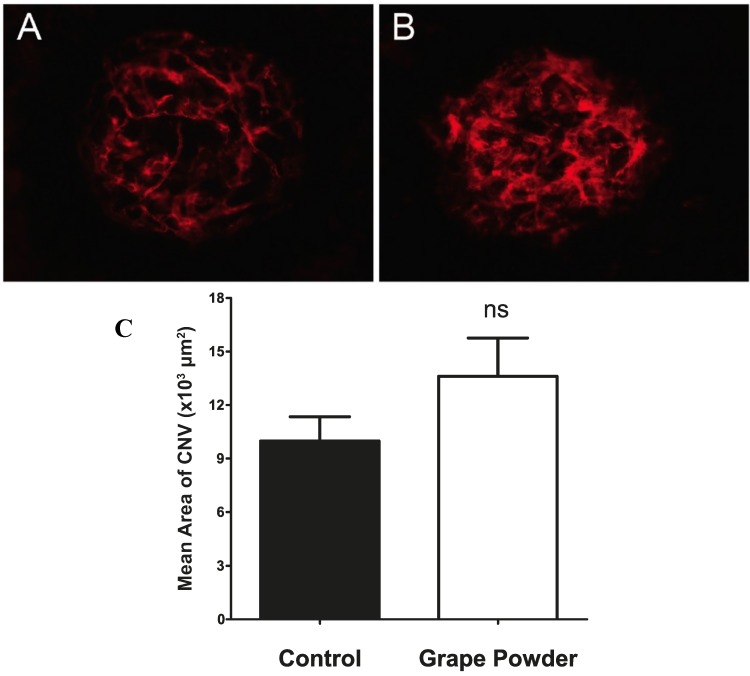
Single daily delivery of reconstituted grape powder enhances blood vessel formation in a mouse model of CNV. Representative sclerochoroidal flat mounts from mice receiving vehicle (**A**) or reconstituted grape powder (**B**) once daily by oral gavage. Treatment began five days prior to the induction of CNV and continued for a total of 16 days. (**C**) Quantification of the data (*p* value = 0.149; n = 18 eyes; ns: not significant).

The concentration of resveratrol in the reconstituted grape powder, 0.7 mg/kg, resulted in a single daily delivery of approximately 0.07 µg per mouse or a dose equivalent to 3.5 µg/kg/day. Similar to the findings obtained with resveratrol, a slight increase in CNV area was observed in animals treated with reconstituted grape powder by daily oral gavage compared to controls (Figure 4A–C). However, the difference was not statistically significant (*p* value = 0.149). The average CNV area for the group receiving reconstituted grape powder was 13,611 ± 2147 µm^2^; n =1 8 eyes, while the control group had an average CNV area of 9984 ± 1364 µm^2^; n = 18 eyes. 

### 2.4. Continuous Delivery of Resveratrol through a Slow Release Osmotic Pump

**Figure 5:**
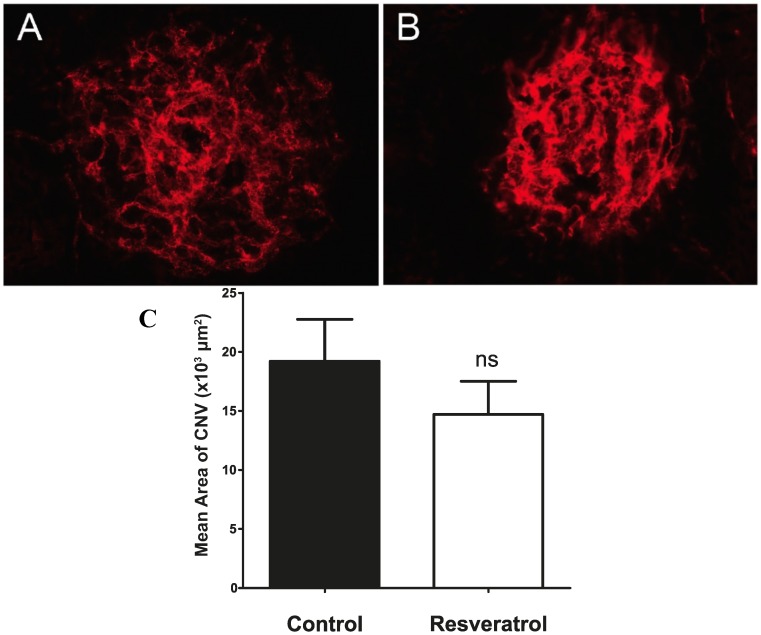
Continuous release of lower levels of resveratrol does not affect new blood vessel formation in a mouse model of CNV. Representative sclerochoroidal flat mounts from mice receiving vehicle (**A**) or resveratrol (**B**) via slow release osmotic pumps. Treatment began five days prior to the induction of CNV and continued for a total of 14 days. (**C**) Quantification of the data (*p* value = 0.322; n = 10 eyes; ns: not significant).

The results from the first experiments indicated that the frequency of delivery of resveratrol or the reconstituted grape powder might be critical for the reduction of CNV. To test this possibility, we implanted slow release osmotic pumps containing either resveratrol or control vehicle. Under these conditions pumps delivered 12 µL/day of a 5 mg/mL resveratrol solution or 60 µg resveratrol/mouse/day, corresponding to a dose of 3.0 mg/kg/day. As shown in Figure 5A–C, the CNV area in mice implanted with slow release osmotic pumps containing resveratrol averaged 14,697 ± 2817 µm^2^; n = 10 eyes.

This value was not statistically different (*p* value = 0.322) from the paired control group (19,205 ± 3561 µm^2^; n = 10 eyes). Owing to the limited compatibility of the osmotic pumps with DMSO and/or ethanol, as well as the limited solubility of resveratrol in aqueous solutions, it wasn’t possible to achieve higher doses of resveratrol using osmotic pumps, therefore, while delivery was constant, resveratrol levels were lower than could be attained either through the drinking supply or by oral gavage.

### 2.5. Resveratrol Causes Dose- and Time-Dependent Growth Inhibition of Choroidal Endothelial Cells in Vitro

**Figure 6:**
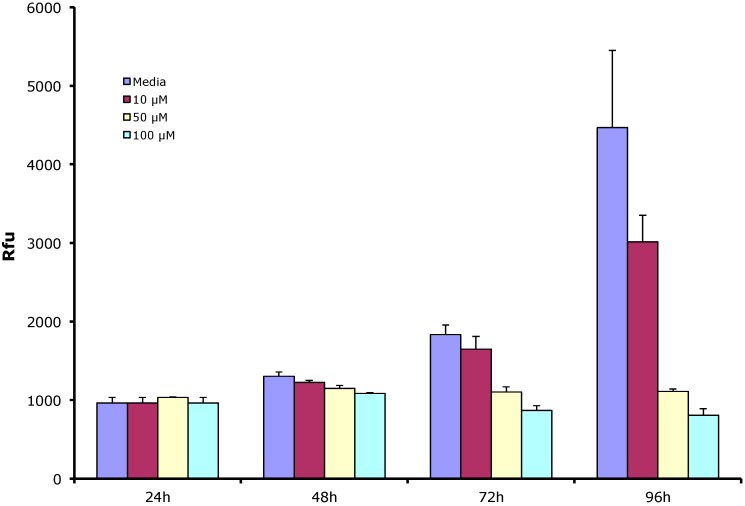
Resveratrol inhibits choroidal endothelial cell growth. Cells were grown in 96-well plates and treated with different concentrations of resveratrol or vehicle control. At 1–4 days, cells were incubated with CellTiter Blue and fluorescence measured.

To assess the anti-proliferative activity of resveratrol, choroidal endothelial cells were exposed to increasing concentrations of resveratrol, and cell viability was measured at different times post-treatment. As illustrated in Figure 6, resveratrol caused a significant decrease in cell viability as a function of both exposure time and concentration. At 96 h post-treatment, the IC_50_ for resveratrol was 26 µM. Resveratrol has been shown in other studies with different cell types to inhibit cell proliferation and induce apoptosis [57,58,59].

### 2.6. Resveratrol Activates p53 in Choroidal Endothelial Cells

**Figure 7:**
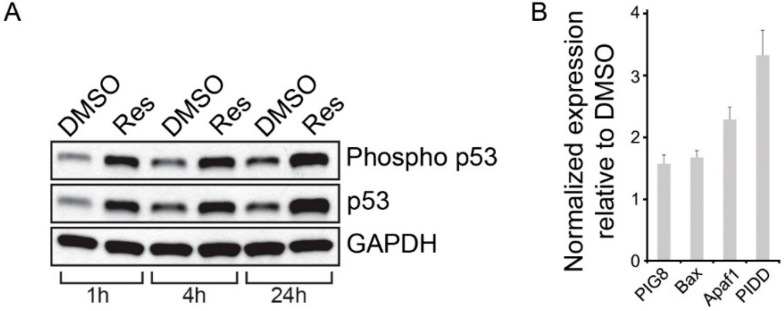
(**A**) Resveratrol increases the expression and phosphorylation of p53 in choroidal endothelial cells. Cells were exposed to 100 µM resveratrol, after which proteins were resolved by SDS-polyacrylamide gel electrophoresis and western blots were stained with specific antibodies. GAPDH served as a control for equal protein loading of the gel. (**B**) RNA from cells treated with 100 µM resveratrol for 6 h was used for qPCR with primers for p53-dependent genes and PolR2a-primers as an internal standard.

p53 is a regulator of the cell cycle, and as such plays a significant role in proliferation, apoptosis and angiogenesis [60]. To determine whether resveratrol activates p53 in choroidal endothelial cells, thereby potentially inhibiting their growth as shown in the previous experiment, cells were treated with resveratrol for different periods of time and p53, both protein and phosphorylated protein levels, were measured by western blots analysis (Figure 7A). Within 1 h of treatment with resveratrol p53 protein levels increased and remained elevated for at least 24 h. The degree of p53 phosphorylation increased concomitantly. To further establish that resveratrol activates p53, choroidal endothelial cells were treated with resveratrol and the transcript levels of several key genes targeted by p53 were measured by quantitative PCR (Figure 7B).

### 2.7. Resveratrol Inhibits the Migration of Choroidal Endothelial Cells in Vitro

A transwell assay was used to determine whether resveratrol could inhibit the migration of choroidal endothelial cells.

**Figure 8:**
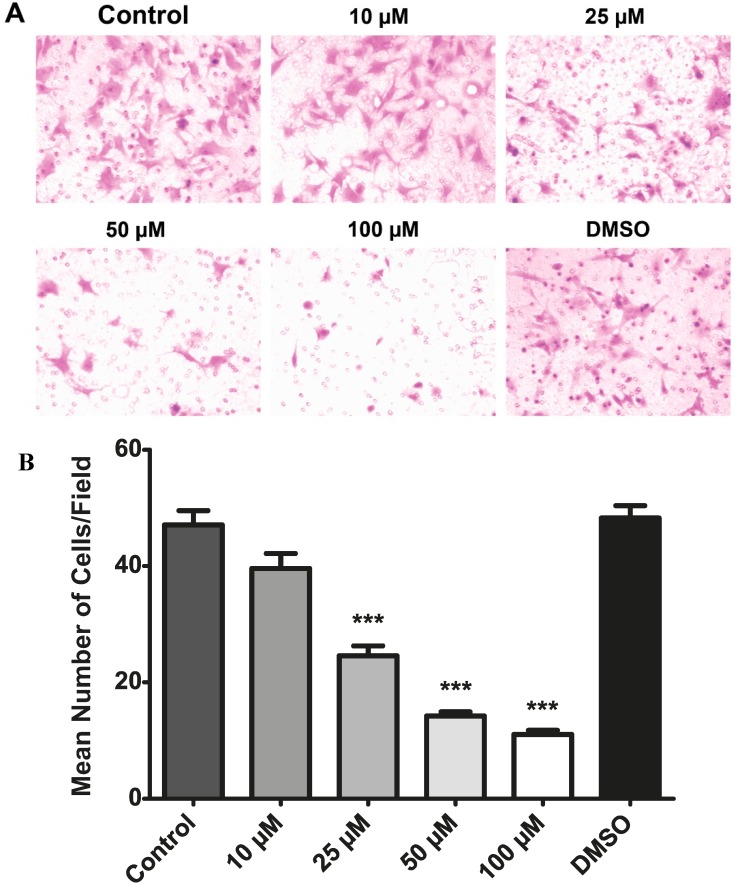
Resveratrol inhibits the migration of choroidal endothelial cells. A transwell assay was utilized to measure the number of cells migrating through a membrane with 8-µm pores as a function of the resveratrol concentration compared to vehicle controls. Cells that traversed the membrane were stained with H&E (**A**) and then counted (**B**). *** *p* ≤ 0.001.

As shown in Figure 8A, cells exposed to medium or medium with DMSO migrated through the 8 µm pores of a membrane and bound to the lower surface coated with fibronectin. Resveratrol decreased the number of cells migrating through the membrane in a dose-dependent manner during the 4 h assay, during which time resveratrol has no measureable effect on choroidal endothelial cell proliferation or apoptosis. The quantitative findings from these experiments are shown in Figure 8B, demonstrating that concentrations of resveratrol as low as 25 µM produce statistically significant inhibitory effects.

### 2.8. Resveratrol Inhibits Akt/Protein Kinase B in Choroidal Endothelial Cells

**Figure 9:**
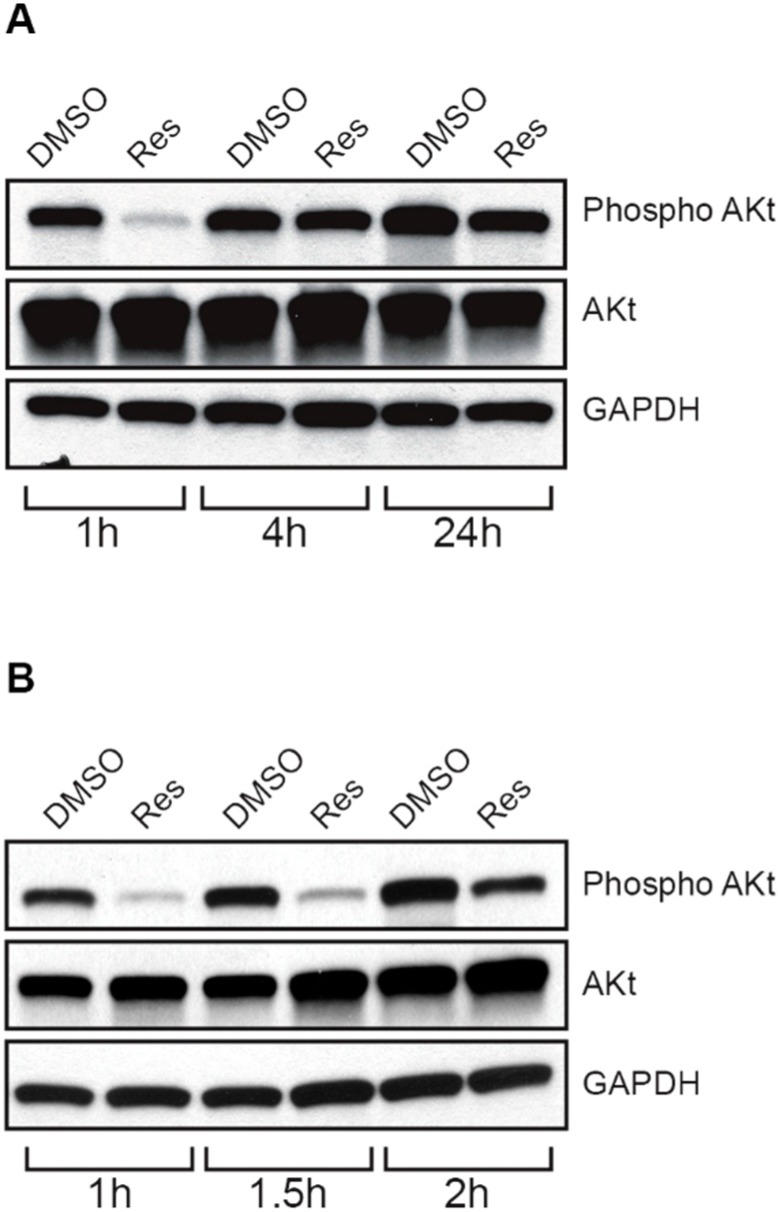
Resveratrol inactivates Akt/protein kinase B in choroidal endothelial cells. (**A**) Cells were exposed to 100 µM resveratrol for different periods of time, after which proteins were resolved by SDS-polyacrylamide gel electrophoresis and western blots were stained with specific antibodies. GAPDH served as a control for equal protein loading of the gel. (**B**) The experiment was repeated over a shorter time course to resolve the period of Akt rephosphorylation.

Resveratrol has been shown to inhibit the migration and invasiveness of cancer cells [61] through the inhibition of Akt/protein kinase B [62], a serine/threonine protein kinase which plays a role during angiogenesis [63]. Therefore, to evaluate the inhibition of choroidal endothelial cell migration, the activity of Akt/protein kinase B was determined after treatment with resveratrol. As shown in Figure 9A, resveratrol inactivated Akt/protein kinase B. Following treatment with resveratrol, choroidal endothelial cells were subjected to western blot analysis using antibodies directed against Akt/protein kinase B and phospho (S473) Akt/protein kinase B. Resveratrol caused a decrease in the phosphorylation of Akt/protein kinase B within 1 h of treatment, while the protein levels of Akt/protein kinase B remain unchanged. As seen in Figure 9B, the re-phosphorylation of Akt/protein kinase B begins approximately 2 h post-treatment in the absence of additional resveratrol.

### 2.9. Resveratrol Inhibits Choroidal Endothelial Cell Tube Formation as an in Vitro Correlate of Angiogenesis

**Figure 10:**
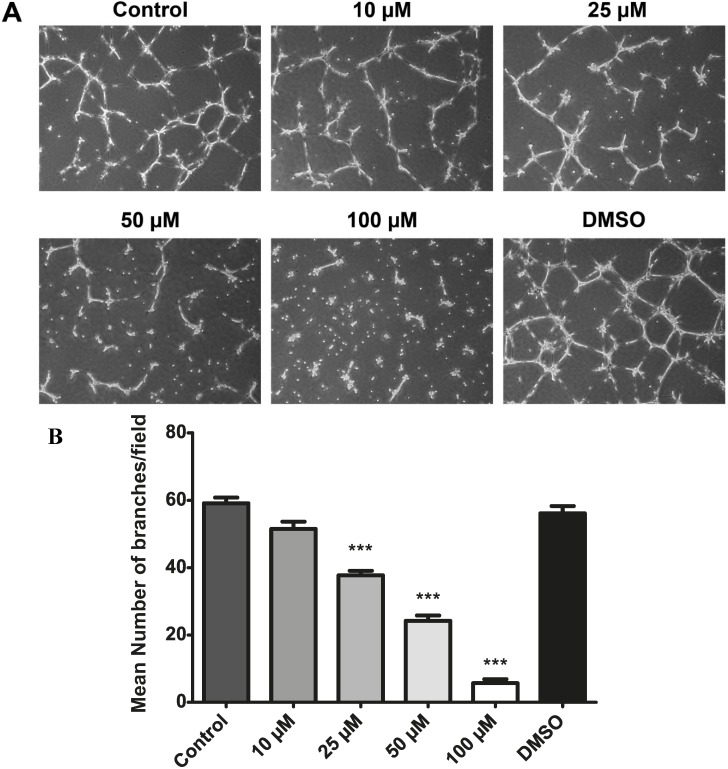
Resveratrol inhibits choroidal endothelial cell branching and network formation. Cells were plated on matrigel in the presence of different concentrations of resveratrol or vehicle controls. Cells were imaged fourteen hours post-treatment (**A**) and the number of branches counted (**B**). *** *p* ≤ 0.001.

Choroidal endothelial cells were plated on matrigel, and the formation of branches and connecting networks was monitored over time as a function of resveratrol treatment (Figure 10). During the treatment period, cells were exposed to serum-free medium to avoid serum constituents that interfere with capillary morphogenesis. While cells exposed to medium or medium containing DMSO produced extensions that branched and formed networks, increasing concentrations of resveratrol inhibited these processes (Figure 10A). Quantitative analysis of the branching activity of choroidal endothelial cells as a function of resveratrol concentration is shown in Figure 10B. The data demonstrate that concentrations of resveratrol as low as 25 µM inhibited the formation of branches, and thereby the ability of choroidal endothelial cells to form networks, events analogous to blood vessel formation.

### 2.10. Resveratrol Increases Cytoplasmic Calcium in Activated Endothelial Cells

**Figure 11:**
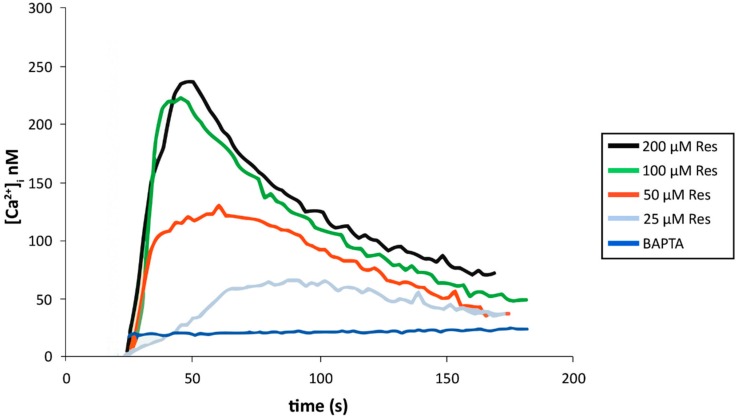
Resveratrol increases cytoplasmic calcium in activated endothelial cells. Cells were loaded with the calcium reporter, fura-2 AM and live imaging was performed using a BD Pathway microscope. Following baseline data collection for the first 25 s, cells were exposed to different concentrations of resveratrol. The average fluorescence output was converted to an average calcium concentration. Pre-incubation with BAPTA-AM, a calcium chelator, abolished the response to resveratrol.

Calcium is perhaps the most ubiquitous, non-degradable second messenger in biology. It plays essential roles during cell division and apoptosis [64]. Resveratrol has been shown to activate early calcium signaling in cancer and muscle cells [58,65], and a portion of the anti-proliferative and pro-apoptotic activities of resveratrol have been tied to this rise in cytoplasmic calcium [58,66]. To determine whether resveratrol elicits similar calcium events in endothelial cells, live cell calcium imaging was performed on a BD Pathway Bioimaging microscopy system with fura-2 loaded cells. As shown in Figure 11, resveratrol caused an increase in cytoplasmic calcium in a dose-dependent manner. Within 5–35 s after exposure to resveratrol, cells exhibited a dose-dependent increase in cytoplasmic calcium, reaching a maximum of 250 nM above baseline levels of calcium approximately 20 s post-treatment. The responses then returned to baseline in a monophasic manner. To verify that these resveratrol-induced changes in calcium detected with fura-2 actually reflect changes in cytoplasmic calcium, cells were first incubated with a cell-permeable calcium chelator, BAPTA-AM, thereby enhancing the buffering capacity of the cells, and the response to resveratrol then was measured using the same live imaging methods. Under these conditions the resveratrol response was abolished (Figure 11).

**Figure 12:**
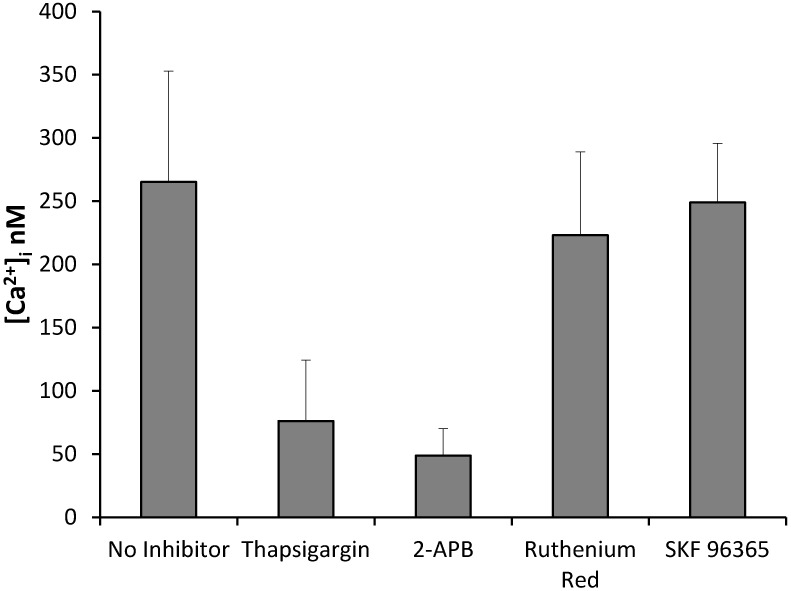
Resveratrol causes the release of calcium from the endoplasmic reticulum of activated endothelial cells. Cells were loaded with fura-2 AM, and live imaging was performed with a BD Pathway microscope. Cells were pre-incubated with different pharmacological agents or vehicle control and then treated with 100 µM resveratrol.

The endoplasmic reticulum (ER) has been shown to be one source of calcium during the resveratrol response in cancer cells [58]. Since thapsigargin is known to deplete ER calcium by inhibiting SERCA, the energy-dependent ER calcium pump, cells were pre-incubated with thapsigargin and then exposed to resveratrol (Figure 12). Upon addition of resveratrol there was a 71% decrease in the amount of calcium released from the ER compared to controls, verifying that the two compounds mobilize the same ER pool of calcium. Additional pharmacological experiments demonstrate that resveratrol causes the release of ER calcium through the IP_3_ receptor (IP_3_R), since pre-incubation with 2-APB, a known inhibitor of the IP_3_R, like thapsigargin reduces the calcium response during the subsequent addition of resveratrol. Agents that block the ryanodine receptor have no effect on the resveratrol-induced release of ER calcium. Likewise, SKF96365, an inhibitor of store-operated channels, has no effect on calcium signaling induced by resveratrol. Collectively, these data are consistent with studies of resveratrol-induced calcium signaling in other cell types.

### 2.11. Discussion

The studies presented here demonstrate that resveratrol and reconstituted grape powder can reduce the growth of new blood vessels in a mouse model of CNV. The anti-angiogenic effect of these compounds is comparable to that obtained with single or multiple intravitreal injections of ranibizumab or bevacizumab observed in a mouse model of subretinal neovascularization [67]. Of significance however, resveratrol has no associated toxicity at the levels used in this study or at considerably higher levels [68,69,70,71], and the anti-angiogenic effects observed here can be achieved without penetrating the eye.

Despite its marginal solubility in water, resveratrol inhibited neovascularization when it was administered throughout the day in the drinking water. In addition to uptake in the stomach and small intestine, this mode of delivery is associated with both sublingual and buccal absorption, allowing resveratrol and grape components to partially escape the first pass effect of metabolism in the small intestine and liver and to enter directly into the blood stream in a free unconjugated form [69,70,71,72]. This may explain why, in addition to the shorter duration of delivery, higher doses of resveratrol delivered only once per day by oral gavage were insufficient to reduce neovascularization. In order to achieve the same plasma level of free resveratrol in humans after receiving 1mg of resveratrol in an alcohol/water solution in the mouth for one minute before swallowing, individuals had to ingest a 250 mg-tablet of resveratrol [69], further reflecting the significance of buccal delivery.

In addition to the route and rate of delivery, the amount of resveratrol also was a determinant factor for efficacy. As shown in these experiments, an oral dose of 25 mg/kg/day of resveratrol delivered over a 24 h timeframe proved sufficient to reduce CNV. In contrast, lower dosage (3 mg/kg/day) of resveratrol alone, delivered over the same timeframe, failed to attain the desired anti-angiogenic response. However, as shown in these studies lower doses of resveratrol could reduce CNV when coupled with other bioactive agents present in grape powder. Delivery of the reconstituted grape powder over the 24 h timeframe through the drinking water was effective in reducing CNV, while the same amount of grape powder delivered by daily oral gavage had no significant effect. Therefore, the rate and route of administration as well as the amount of resveratrol and other bioactive grape components determine the anti- or pro-angiogenic outcome from treatment.

The anti-angiogenic effect of resveratrol observed in these experiments of CNV is consistent with other models of ocular disease. For example, resveratrol inhibited pathological angiogenesis in the retina of very low-density lipoprotein receptor knock-out (Vldlr^−/−^) mice [55] and also blocked diabetes-induced early vascular lesions [56]. These anti-angiogenic responses to resveratrol also are consistent with observations that resveratrol can decrease VEGF expression [73,74,75], as well as inhibit the *secretion* of VEGF from retinal pigment epithelial cells [76]. Reduced levels of VEGF in turn undermine the proliferation of choroidal endothelial cells [77], presumably decreasing neovascularization in pre-clinical models of CNV.

A single daily delivery of resveratrol by oral gavage recently was shown to reduce neovascularization in a laser-induced rodent model of CNV [78]. These results contrast with our findings, whereby intragastric delivery failed to elicit an anti-angiogenic response. Differences in experimental design regarding the length of resveratrol treatment may have contributed to the different findings. Along these lines, more prolonged pre-treatment with resveratrol through intragastric delivery could have improved the anti-angiogenic response in our study, comparable to the improvement we measured with ad libitum water intake. Regardless of these differences, the recent report [78] and our findings support the observation that resveratrol treatment can potentially improve retinal structure and function in patients with AMD [79].

Studies presented here also demonstrate that resveratrol increases p53 activity, which could explain in part the inhibitory effect of resveratrol measured in our *in vitro* viability assays of choroidal endothelial cells. This is not to say that the activation of p53 is the only molecular event underlying the anti-proliferative and pro-apoptotic effects of resveratrol, since we and others have shown that resveratrol can activate many critical pathways, including the intrinsic apoptotic pathway, as well as an endoplasmic reticulum stress response, and additional transcriptional events [22,57,58,59]. Resveratrol also can modulate the formation of new blood vessels through the aryl hydrocarbon receptor and its regulation of VEGF expression in the endothelium [80]. While the multi-faceted mechanisms of action elicited by resveratrol make it an attractive product for the treatment of disparate diseases, that same diversity of action makes it very difficult to establish the relative significance of any one pathway.

The activation of p53 by resveratrol was supported by the observation of increased p53-dependent transcripts, encoding several pro-apoptotic gene products associated with different cellular compartments, including the endoplasmic reticulum, mitochondria and the cytoplasm. In the same studies p21 levels were found to be low and unresponsive to treatment with resveratrol. This may indicate that p53 primarily plays a pro-apoptotic role rather than an anti-proliferative one, at least in our *in vitro* studies of choroidal endothelial cell viability.

We also found that resveratrol inhibited Akt/protein kinase B activity in choroidal endothelial cells, consistent with the anti-migratory properties of resveratrol observed in our *in vitro* studies. Resveratrol also inhibited branching and network formation by choroidal endothelial cells, an *in vitro* correlate of angiogenesis. Together, it’s likely that Akt/protein kinase B and p53 contribute to the overall anti-angiogenic response to resveratrol seen in animals with laser-induced CNV. It’s also important to recognize that calcium signals can regulate a number of these pro-apoptotic and anti-angiogenic pathways, and here we demonstrate that resveratrol activates the release of ER calcium in endothelial cells as seen in other cell types [58]. As a consequence, the development of calcium mimetics based on studies of natural products such as resveratrol could provide a novel strategy for the treatment of neovascular diseases.

Regarding the administration of reconstituted grape powder, our observations also are consistent with previous studies, in which anti-angiogenic effects of grape seed proanthocyanidins were mediated through the down-regulation of both VEGF and angiopoietin signaling [53,81]. Further, among the flavonols present in grapes, the amount of quercetin is roughly 100-fold higher than resveratrol and has been shown to inhibit the proliferation and migration of choroid-retina endothelial cells [82] and inhibit choroidal neovascularization in an animal model [83]. In contrast, the circulating metabolites of quercetin have the opposite effect on angiogenesis [84], therefore the outcome of treatment may depend on the subtle balance between the parent compound and it metabolites. As with resveratrol, we found in our study that administration of grape powder through the ad libitum intake of water inhibited neovascularization in the laser-induced model of CNV, while single daily delivery by oral gavage slightly enhanced angiogenesis. Similar to resveratrol, it appears that grape powder can induce either anti- or pro-angiogenic responses in a CNV model. The efficacy of grape powder therefore likely depends upon the bioavailability of a complex array of active components and metabolites.

Due to the variable pharmacokinetics of resveratrol associated with different routes of administration, and the problem of first pass metabolism by liver and small intestine enzymes, consumption of resveratrol by humans may lead to variable plasma levels of the free unconjugated compound. However, as shown in the studies presented here, the route and duration of administration as well as the overall amount of resveratrol need to be tightly controlled in order to elicit the desired anti- or pro-angiogenic response appropriate for the treatment of a particular disease. For example, we observed a small but statistically significant *increase* in choroidal neovascularization upon treatment with a single, daily dose of resveratrol by oral delivery. This finding may reflect the difficulty of controlling neovascularization when consuming resveratrol. Therefore, while resveratrol and other grape components are excellent prototypes, having the potential to reduce the extent of visually harmful blood vessels associated with the exudative form of AMD, it may be beneficial to formulate drugs based on the structure of resveratrol that provide consistent bioavailability and ease of delivery for the safe and effective treatment of AMD. Further, given the preventive properties of resveratrol [22], such drugs may have the added advantage of being able to intervene during the progression of early- to late-stage disease.

## 3. Experimental Section

### 3.1. Materials

Resveratrol was purchased from Cayman Chemical Company (Ann Arbor, MI, USA). A defined grape powder was obtained through the California Table Grape Commission (Fresno, CA, USA). Rat anti-intercellular adhesion molecule 2 antibodies were obtained from BD Biosciences (San Jose, CA, USA). Cy-3 anti-rat secondary antibody was purchased from Jackson ImmunoResearch Laboratories, Inc. (West Grove, PA, USA). Mouse antipan-Akt (clone C67E7), rabbit anti-phospho (S473) Akt (clone D9E), and mouse anti-glyceraldehyde 3-phosphate dehydrogenase (GAPDH) antibodies were obtained from R&D Systems (Minneapolis, MN, USA). Rabbit anti-p53 (clone 7F5) and rabbit anti-phospho (S15) p53 (clone 16G8) antibodies were obtained from Cell Signaling Technology, Inc. (Danvers, MA, USA). Primary mouse choroidal endothelial cells were derived as previously described [78]. Osmotic pumps (Alzet model 2002) were obtained through the Durect Corporation (Cupertino, CA, USA).

### 3.2. Treatment Groups

All animal experiments were performed in accordance with the Association for Research in Vision and Ophthalmology guidelines for the Use of Animals in Ophthalmic and Vision Research and in accordance with an animal protocol approved by the University of Wisconsin School of Medicine and Public Health Animal Care and Use Committee. All caretakers and laboratory staff followed standards set by the Association for the Assessment and Accreditation of Laboratory Animal Care International.

Six-week-old female C57BL/6J mice, an inbred strain from Jackson Laboratory, were used in these studies. Animals were housed on a 12-h light-dark cycle, with food and water provided *ad libitum.* Mice were randomized into 11 groups. First study: the control group consisted of five animals which received water ad libitum for 16 days and a treatment group consisting of five animals which received water containing 0.1 mg/mL resveratrol *ad libitum* for 16 days. Laser treatment began five days following treatment. Second study: the control group consisted of five animals which received water *ad libitum* for 24 days, a treatment group consisting of five animals which received water containing 0.1 mg/mL resveratrol *ad libitum* for 24 days, and a second treatment group consisting of five animals which received water containing 20 mg/mL grape powder *ad libitum* for 24 days. Laser treatment began twelve days following treatment. Third study: the control group consisted of ten animals which received 100 µL of Neobee oil daily by oral gavage for 16 days, and a treatment group of ten animals which received 1 mg resveratrol (50 mg/kg dose) in 100 µL Neobee oil daily by oral gavage for 16 days. Laser treatment began five days following treatment. Fourth study: the control group consisted of 10 animals which received 500 µL of water daily by oral gavage for 16 days, and a treatment group of ten animals which received 100 mg grape powder reconstituted in 500 µL water daily by oral gavage for 16 days. Laser treatment began five days following treatment. Fifth study: the control group consisted of five animals implanted with a pump loaded with 200 µL of vehicle (50% DMSO and 15% EtOH) delivered at a rate of 0.5 µL/h for 14 days, and a treatment group of five animals implanted with a pump loaded with 1 mg resveratrol per 200 µL vehicle delivered at a rate of 0.5 µL/h (2.5 µg resveratrol per hour) for 14 days. Laser treatment began five days following treatment. A total of four mice had to be eliminated from the studies due either to the development of laser-induced bilateral vitreous hemorrhages or other health-related issues; their removal is reflected in the n value reported for each of the study groups.

### 3.3. Implantation of Alzet Pumps

Slow release osmotic Alzet pumps were loaded with either vehicle or resveratrol, weighed, and as previously described each was then surgically implanted subcutaneously on the flanks of mice [36]. At the end of the study, after euthanizing the animals, the implanted pumps were removed, weighed and further evaluated for any residual loaded material to ensure correct functioning of the pumps during the study.

### 3.4. Laser-Induced CNV

The rupture of Bruch’s membrane in C57BL/6J mice was accomplished using a laser. Briefly, mice were anesthetized by intraperitoneal injection of ketamine hydrochloride (75 mg/kg) and xylazine (7.5 mg/kg). Pupillary dilation was achieved using tropicamide 1% eye drops. Three bursts of a 532 nm diode laser (75 mm spot size, 0.1 s duration, 120 mW) were delivered to each retina in the 9-, 12-, and 3-o'clock positions. The procedure was performed with the slit lamp delivery system of an OcuLight GL diode laser (Iridex, Mountain View, CA, USA) and a round glass coverslip as a contact lens to view the retina [85]. After euthanizing the mice, eyes were enucleated and fixed in 4% paraformaldehyde at 4 °C for 2 h. After transferring the eyes to phosphate-buffered saline (PBS), they were sectioned at the equator to obtain posterior sclerochoroidal eyecups. Following incubation in blocking buffer (50% fetal calf serum, 20% normal goat serum and 0.01% Triton-X-100 in PBS) for 1 h at room temperature, eyecups were incubated with anti-intercellular adhesion molecule-2 antibodies (1:500 in PBS containing 20% fetal calf serum, 20% normal goat serum and 0.01% Triton-X-100) overnight at 4 °C. Eyecups were then washed with PBS and incubated with a Cy-3 labeled secondary antibody (1:500 in PBS containing 20% fetal calf serum, 20% normal goat serum and 0.01% Triton-X-100) for 2 h. Afterwards, eyecups were flattened onto a glass slide through 5 to 6 relaxing radial incisions and mounted with VectaMount AQ (Vector Laboratories, Burlingame, CA, USA). Samples were visualized using the 20× objective of an epifluorescent compound microscope fitted with appropriate excitation and emission filters (AxioPhot, Zeiss, Germany). Images were captured using a digital camera (AxioCam, HRm; Zeiss, Oberkochen, Germany) and compatible software (AxioVision 4.8.2, Zeiss). To quantify the total area of CNV associated with each laser spot, ImageJ free-ware was used.

### 3.5. Statistical Analysis of Animal Studies

Means and standard deviations of the CNV area in each group were calculated. Statistical differences between each treatment group and their respective control group were determined using an unpaired two-tailed *t*-test. *p* values less than 0.05 were considered significant.

### 3.6. In Vitro Cell Viability

Choroidal endothelial cells were derived from Immorto mice (H-2K(b)-ts-A58(+/+) using procedures established in our laboratory [86]. Cells were grown in 96-well plates for 2 days and then incubated with different concentrations of resveratrol or DMSO in serum-containing medium for an additional 1–4 days. CellTiter Blue was added each day to a portion of the cells according to the manufacturer’s instructions (Promega, Madison, WI, USA). Fluorescence was measured at excitation/emission wavelengths of 560/590 nm with a fluorescence plate reader.

### 3.7. Western Blot Analysis

Cells were treated with DMSO or resveratrol in serum-containing medium for designated periods and washed twice with Hank’s Balanced Salt Solution (HBSS) before their collection by scraping in HBSS supplemented with protease and phosphatase inhibitors. Following centrifugation, equal amounts of protein in SDS-solubilization solution were loaded onto 10% polyacrylamide gels, transferred to polyvinylidene fluoride membrane and stained with antibodies. Antibody binding was detected using an ECL kit from Amersham GE Healthcare (Piscataway, NJ, USA).

### 3.8. Cell Migration Assay

Transwell inserts (8-µm pore size, 6.5-mm membrane; Costar, Lowell, MA, USA) were coated with PBS containing fibronectin (2 µg/mL) on the bottom side of the membrane at 4 °C overnight. After washing with PBS, inserts were blocked in PBS containing 1% BSA for 1 h at room temperature. Choroidal endothelial cells were detached with trypsin and suspended in serum-free DMEM medium. Different concentrations of resveratrol or DMSO alone were added to 1 × 10 ^5^ cells/0.1 mL. Cell suspensions were kept on ice for 20 m and then added to the top of the inserts. The inserts were placed in a 24 well plate containing 0.5 mL of serum-free medium and incubated for 4 h at 33 °C. Cells then were fixed with 2% paraformaldehyde for 10 min at room temperature, stained with hematoxylin and eosin (H& E), and the inserts were mounted on a slide. The number of cells that migrated through the membrane was determined by counting 10 high- power fields (×200). Each experiment employed triplicate samples.

### 3.9. Capillary Morphogenesis Assay

Tissue culture plates (35 mm) were coated with 0.5 mL matrigel (9 mg/mL, BD Bioscience) and incubated at 37 °C for at least 30 min. Choroidal endothelial cells were removed with trypsin- EDTA, washed with DMEM containing 10% FBS, and suspended in serum-free DMEM medium containing different concentrations of resveratrol. Cells (2 × 10^5^/2 mL) were kept on ice for 20 min and then applied to the matrigel-coated plates, incubated at 37 °C, and photographed after 14 h with a Nikon microscope in a digital format. For quantitative assessment of the data, the mean number of branch points was determined by counting the branch points in five high-power fields (×100).

### 3.10. Calcium Imaging

Briefly, endothelial cells were loaded with fura-2-AM for 30 min at 37 °C followed by an additional incubation for 30 min at room temperature. Cells were subsequently imaged on a BD Pathway microscope following a standard protocol [58]. The fura-2 loaded cells were alternately excited at 340 and 380 nm and emission was monitored at 510 nm. Drugs were added following a baseline collection, and the average fluorescence output was converted to average calcium concentration via the Grynkiewicz equation [87].

## 4. Conclusions

Resveratrol and reconstituted grape powder can reduce harmful blood vessels associated with models of the exudative form of age-related macular degeneration, a leading cause of blindness in the industrialized world. Resveratrol activates calcium signals which initiate a number of anti-proliferative and pro-apoptotic pathways that minimize endothelial cell growth and migration. Resveratrol also interferes with the ability of endothelial cells to form networks.

In addition to the effects of resveratrol on VEGF expression reported previously, the current study demonstrates that p53 and Akt/protein kinase B also are modulated by resveratrol, likely contributing to its pro-apoptotic and anti-migratory properties. Finally, to accurately establish the bioavailability of resveratrol needed to attain the desired anti-angiogenic response in patients with neovascular age-related macular degeneration, modifications of the parent compound would be useful that minimize its rapid metabolism and improve its solubility, thereby allowing more controlled drug delivery through an intravenous route.
